# The interplay between non-esterified fatty acids and bovine peroxisome proliferator-activated receptors: results of an *in vitro* hybrid approach

**DOI:** 10.1186/s40104-020-00481-y

**Published:** 2020-08-11

**Authors:** Sebastiano Busato, Massimo Bionaz

**Affiliations:** Department of Animal and Rangeland Sciences, Oregon State University, Corvallis, OR USA

**Keywords:** Albumin, Blood serum, Bovine, Gene reporter, Hepatocytes, Lipoprotein lipase, Mammary cells, Non-esterified fatty acids, Peroxisome proliferator-activated receptor

## Abstract

**Background:**

In dairy cows circulating non-esterified fatty acids (NEFA) increase early post-partum while liver and other tissues undergo adaptation to greater lipid metabolism, mainly regulated by peroxisome proliferator-activated receptors (PPAR). PPAR are activated by fatty acids (FA), but it remains to be demonstrated that circulating NEFA or dietary FA activate bovine PPAR. We hypothesized that circulating NEFA and dietary FA activate PPAR in dairy cows.

**Methods:**

The dose-response activation of PPAR by NEFA or dietary FA was assessed using HP300e digital dispenser and luciferase reporter in several bovine cell types. Cells were treated with blood plasma isolated from Jersey cows before and after parturition, NEFA isolated from the blood plasma, FA released from lipoproteins using milk lipoprotein lipase (LPL), and palmitic acid (C16:0). Effect on each PPAR isotype was assessed using specific synthetic inhibitors.

**Results:**

NEFA isolated from blood serum activate PPAR linearly up to ~ 4-fold at 400 μmol/L in MAC-T cells but had cytotoxic effect. Addition of albumin to the culture media decreases cytotoxic effects of NEFA but also PPAR activation by ~ 2-fold. Treating cells with serum from peripartum cows reveals that much of the PPAR activation can be explained by the amount of NEFA in the serum (R^2^ = 0.91) and that the response to serum NEFA follows a quadratic tendency, with peak activation around 1.4 mmol/L. Analysis of PPAR activation by serum in MAC-T, BFH-12 and BPAEC cells revealed that most of the activation is explained by the activity of PPARδ and PPARγ, but not PPARα. Palmitic acid activated PPAR when added in culture media or blood serum but the activation was limited to PPARδ and PPARα and the response was nil in serum from post-partum cows. The addition of LPL to the serum increased > 1.5-fold PPAR activation.

**Conclusion:**

Our results support dose-dependent activation of PPAR by circulating NEFA in bovine, specifically δ and γ isotypes. Data also support the possibility of increasing PPAR activation by dietary FA; however, this nutrigenomics approach maybe only effective in pre-partum but not post-partum cows.

## Introduction

The period comprised between 21 days before and after parturition, commonly referred to as “transition period” or “peripartum”, is widely regarded as one of the most metabolically challenging events in the life of a dairy cow [1, 2]. The large increase in energy requirements, combined with a general decrease in dry matter intake around calving, is translated in a state of negative energy balance (NEB), sustained during the early post-partum [3, 4]. The direct implications of this phenomenon are usually understood in terms of changes in glucose and lipid metabolism, where the hydrolysis of triacylglycerol (TAG) in the adipose tissue effects a sharp increase in circulating non-esterified fatty acids (NEFA), generally measurable in the order of > 0.5 mmol/L during the early post-partum [5, 6].

An increase in circulating NEFA is often an accurate indicator of NEB in dairy cows [7]. As the concentration of NEFA in the blood increases, so does their uptake by the liver, where they can be either oxidized or repackaged into TAG [8]; thus, the development of hepatic lipidosis might occur, causing additional strain on this already overexerted organ, and impairing its function [9]. Furthermore, NEFA have cytotoxic effects in several cell types including leukocytes [4], as well as contributing to overall oxidative stress via mitochondrial β-oxidation [10], increase rates of ketogenesis [11], and have a negative impact on the immune system, typical of early post-partum dairy cows [12]. All these factors, combined with presence of inflammatory-like conditions that stimulate the hepatic acute response (i.e., increase circulating haptoglobin and decrease of albumin and other negative acute phase proteins) [13], result in the overrepresentation of diseases, which about half of early post-partum cows have to endure [14].

Studies conducted in energy modulation during the transition period have highlighted that cows whose feed had been restricted in the dry period had lower postpartum release of NEFA, when compared to cows that were overfed during the same time frame [15]. This suggests a potential role of higher NEFA prepartum in priming the liver to face incoming metabolic challenges, enhancing fatty acids (FA) oxidation and reducing esterification rates [16]. Chiefly, large-scale transcriptomic comparisons of cows overfed vs. restricted in the dry period confirms this hypothesis, unveiling a major role of the peroxisome proliferator-activated receptors (PPAR), especially PPARα [17].

PPAR are a family of ligand-dependent nuclear receptors comprised of three related isotypes: PPARα, highly expressed in liver, PPARβ/δ expressed ubiquitously among tissues, and PPARγ expressed at high levels in the adipose tissue [18]. Upon binding of a suitable ligand, PPAR isotypes locate a 13-nucleotide PPAR response element (PPRE) in the genome, to which they subsequently bind, recruit the transcriptional machinery, and induce the expression of downstream target genes [19]. All PPAR are capable of utilizing FA as ligands and modulate the expression of genes involved in inflammation and glucose and lipid metabolism [20].

Whilst the biology of the peripartum dairy cow has been subject of extensive investigation, the NEFA- and dietary FA-transcriptome interaction in bovine is still a relatively uncharted frontier. While the plausible connection between high circulating NEFA and PPAR activity in ruminants has been previously discussed [18], present studies have failed to demonstrate a direct causal link. Data in mice revealed an increase in expression of PPAR target genes as a response to fasting, when circulating NEFA are high [21]. Increase of PPAR activity as a result of hydrolysis of dietary very low density lipoproteins (VLDL), but not plasma NEFA, were reported in mice [22]. Another study detected a direct correlation between circulating NEFA and expression of PPARβ/δ target genes [23], but a direct causal link between the two was not established. In ruminants an increase in expression of putative PPAR target genes coding for proteins involved in FA and glucose metabolism was observed in the early postpartum [24] when NEFA concentration reaches its peak in plasma.

Present data in non-ruminants and ruminants allowed to propose a hypothetical model where activation of PPAR isotypes by FA, as circulating NEFA or the ones released from circulating lipoproteins mostly of dietary origin, as well as the products of lipolysis that can activate PPAR locally in the adipose tissue [25], can aid dairy cows to transition from pregnancy to lactation [18]. In particular, activation of PPARα and PPARδ by NEFA and dietary FA could increase oxidation of FA in the liver and muscle, while activation of PPARγ could increase insulin sensitivity, reducing adipocytes lipolysis (i.e., NEFA concentration), and increase utilization of FA for milk fat synthesis [18].

Considering the possible agonistic effect of NEFA and dietary FA on PPAR isotypes, there is a need to clearly elucidate whether those modulate PPAR isotypes, to which extent, and through which of the three PPAR isotypes. Those data are important for application of nutrigenomics in dairy cows, as previously argued [18].

In this study, we hypothesize that high circulating NEFA of post-partum cows and serum FA released by lipoprotein lipase (i.e., most dietary FA) activate PPAR isotypes. We elucidate this causality through a hybrid* in vivo-in vitro* model, where immortalized mammary, hepatic and endothelial bovine cells are exposed to natural occurring concentration of circulating NEFA by being cultured in serum from pre- and post-partum cows. Further, we aim to show which PPAR isoform responds more actively to serum NEFA and whether FA released from circulating lipoproteins can further activate PPAR compared to serum alone.

## Materials and methods

### Animals, sampling and diet

Experimental procedures used in this study were approved by the Institutional Animal Care and Use Committees (IACUC) of Oregon State University (protocol# 4894). Serum samples were obtained from the five primiparous Jersey cows utilized in the control group of the study by Jaaf et al. [26]. Preprandial blood samples (~ 20 mL per animal) were drawn from the jugular vein the morning of − 40 ± 6, − 10 ± 2 and + 10 ± 1 day relative to parturition or day in milk (DIM) in Vacutainer blood collection tubes without anti-coagulants (366430, Becton, Dickinson and Company, NJ, USA) as this can negatively affect cell viability [27] and allowed to clot at room temperature for no less than 30 min. The serum was separated by centrifugation for 15 min at 1500×*g* at room temperature, aliquoted and stored at − 20 °C until the time of the experiment.

### NEFA isolation and quantification

NEFA were isolated from 5 mL of each serum sample, following the method by Contreras et al. [28] with few modifications. Briefly, serum samples were mixed with a 1:1 hexane:ethanol (n-Hexane, HX0295–6, OmniSolv, MA, USA; Ethanol, 3916EA, Decon Labs, PA, USA), vortexed for 10 min, and separated by centrifugation for 10 min at 2095×*g* at room temperature. The top hexane layer was collected and flowed using a negative pressure of ~ 10 PSI through a 1 g/ 6 mL Strata aminopropyl (NH_2_) SPE column (8B-S009-JCH, Phenomenex, CA, USA), pre-conditioned twice with 6 mL of hexane. The neutral lipid fraction of the sample was eluted using chloroform:2-propanol (2:1, Chloroform, 9175–02, J.T. Baker, PA, USA; isopropyl alcohol, BDH1174-4LP, VWR, USA), and the NEFA fraction using 2% acetic acid (V193–05, Macron, PA, USA) in diethyl ether (106-1 L, Honeywell, USA). Eluates were evaporated under gentle flow of nitrogen gas and resuspended in DMSO (D2438, Sigma-Aldrich Co, MO, USA), obtaining a 100-fold concentrated stock (10 μL of DMSO/mL of serum). NEFA in serum and isolated NEFA were measured using a HR Series NEFA-HR(2) diagnostic kit (FUJIFILM Wako Diagnostics USA, VA, USA) according to manufacturer’s instructions. A five-point standard curve was used to calculate NEFA concentration in the samples. Average NEFA recovery, measured by diluting the isolated samples 100-fold in DMSO and comparing it to the molarity in the serum sample, was 77.90% ± 4.42%.

### Cell culture and treatments

Immortalized bovine mammary alveolar cells (MAC-T, Cellosaurus accession CVCL_U226) were allowed to grow in vented T75 flasks (25-209, Olympus Plastics, Genesee Scientific, CA, USA) in DMEM (25-500, GenClone, Genesee Scientific, CA, USA), supplemented with 10% FBS, 1% penicillin/streptomycin (25-512, GenClone) and 0.3% Amphotericin B (25-541, GenClone). Immortalized bovine fetal hepatocytes (BFH-12, Cellosaurus accession CVCL_JQ51, Gleich et al. [29]) were provided by Dr. Herber Fuhrmann (University of Leipzig, Leipzig, Germany) and were grown in vented T75 flasks in Williams’ E Medium (A1217601, Gibco, Thermo Fisher Scientific, MA, USA) containing 5% FBS, 1% penicillin/streptomycin, 1% GlutaMAX supplement (35050061, Gibco), 100 nmol/L dexamethasone (AAA17590, Alfa Aesar, MA, USA) and 0.2 U/mL insulin (I6634, Sigma-Aldrich). Bovine pulmonary artery endothelial cells (BPAEC, Cellosaurus accession CVCL_4130) were a gift from Dr. Adam Higgins (Oregon State University, OR, USA), and were cultured in DMEM in vented 75 cm^2^ flasks supplemented with 10% FBS, 1% penicillin/streptomycin and 0.3% Amphotericin B. The cell lines were selected as a model for the bovine mammary gland, liver, and endothelial cells; culture media choices and environmental conditions reflect the protocols developed in each original publication. Most of the experiment were carried out with MAC-T and BFH-12, being mammary and liver tissue critical in peripartum cows.

All cells where cultured for at least 3 passages before the beginning of the experiment. PPAR antagonists used in this experiment were the following: for PPARα, GW-6471 (9453, CAS# 880635-03-0, BioVision incorporated, CA, USA); for PPARδ, GSK-3787 (3961/10, CAS# 188591-46-0, Tocris, Bio-Techne Corporation, MN, USA); for PPARγ, GW-9662 (70785, CAS# 22978-25-2, Cayman Chemicals, MI, USA). Optimal antagonist concentration was determined in MAC-T and BFH-12 using a 4-point linear dilution where the lowest concentration was set at 500 nmol/L, and the highest was set at double the typical concentration found in the literature. For both GW-6471 and GW-9662, the four concentrations used were 500 nmol/L, 33.67 μmol/L, 66.83 μmol/L and 100 μmol/L. For GSK-3787, the concentrations were 500 nmol/L, 3.37 μmol/L, 6.68 μmol/L and 10 μmol/L(Suppl. Figure 1).

### Cell viability assay

Cell viability was calculated through fluorescent imaging using Hoescht 3342 stain (H3570, Molecular Probes, OR, USA) to image cell nuclei and propidium iodide (P1304MP, Molecular Probes) to identify apoptotic/necrotic cells. Cells were imaged using a Leica DMI6000 fully automated inverted microscope (Leica Microsystems, IL, USA) coupled with a SOLA light engine (Lumencor, Beaverton, OR, USA), and blue (long pass set for SOLA, band pass 405–425, Leica Microsystems) and green (I3, band pass 450–490) fluorescent filters. Acquired images were analyzed using the open-source software CellProfiler [30], and viability was calculated as the ratio of PI-stained cells over Hoescht-stained cells, while cellular proliferation was estimated using the number of Hoescht-stained cells (nuclei).

### Plasmid, transfection and luciferase assay

PPRE X3-TK-luc (Addgene plasmid # 1015) [31] a plasmid containing a PPRE-driven luciferase reporter, was used as a measure of PPAR activity. The plasmid pRL-TK (E2231, Promega, WI, USA), encoding *Renilla reniformis* luciferase guided by a minimal thymidine kinase promoter, was co-transfected to normalize the luciferase (i.e., to account for variation in number of cells, transcriptional activity, and/or transfection efficiency). All cell lines were transferred to either 96 well plates (14000 cells/well) or 384 well plates (4,000 cells/well) and, after adhesion to the culture vessel, the plasmid mixture (40:1 ratio luciferase:renilla) was transfected using Lipofectamine 3000 (L3000015, Thermo Fisher Scientific, MA, USA) as previously described [32]. At 12 h post-transfection, the culture media was replaced with either serum or fresh culture media at 80 μL/well in 96-well plates and 40 μL/well in 384-well plates. Agonists, antagonists and additional treatments were added using the D300e Digital Dispenser (HP Inc., CA, USA). All treatments, including control, where normalized to the vehicle (DMSO) by the dispenser (between 0.5% and 1%, depending on the experiment). Plates were assayed at 24 h post-treatment using Dual-Glo® Luciferase Assay System (E2920, Promega, WI, USA), and read in a Synergy HTX Multi-Mode Reader (BioTek, VT, USA). Integration time was 5 s/well, with the gain set at 200.

### Statistical analysis

All gene reporter experiments were analyzed with a generalized linear model, using the GLM procedure of SAS (version 9.4, SAS Institute Inc., NC, USA). Treatment means were separated using Fisher’s least significant difference pair-wise comparisons. To determine the effect of NEFA concentration on PPAR activation, NEFA molarity was used as an explanatory variable. The most suitable model was selected based on the adjusted R^2^ of all tested models. For all other experiments, treatments were set as the fixed effect. Significance was set at *p* ≤ 0.05.

## Results

### Isolated NEFA activate PPAR in a dose-dependent fashion but are cytotoxic

**Fig. 1:**
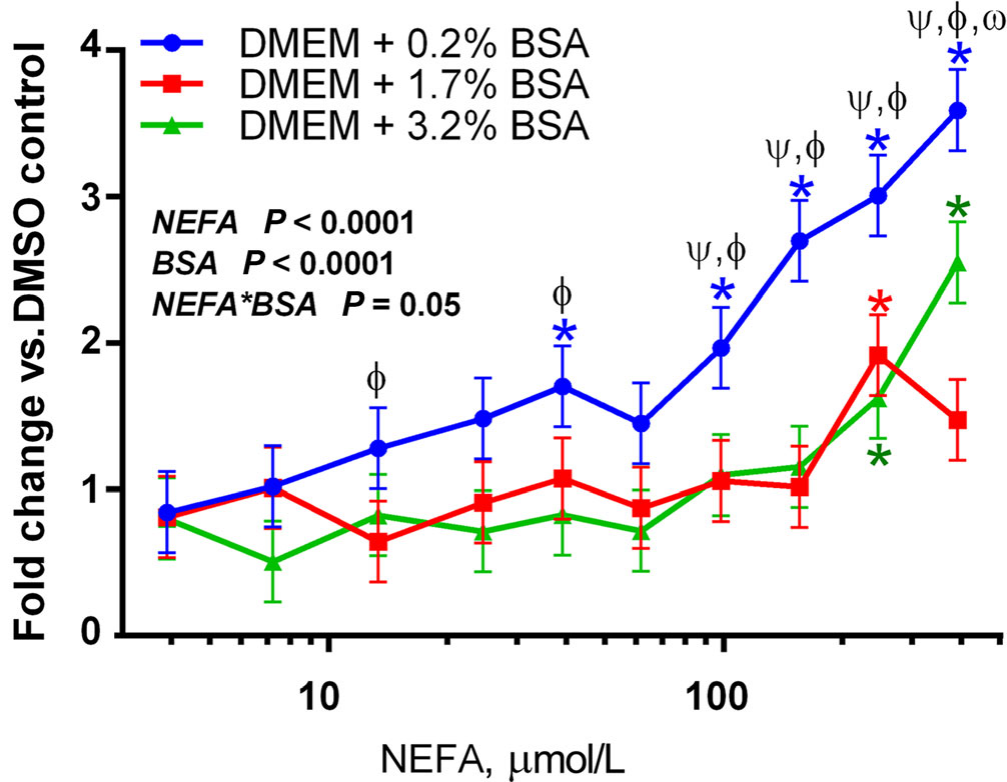
Activation of PPAR by NEFA isolated from a pooled postpartum serum sample of 5 Jersey cows. NEFA were diluted in culture medium either without additional albumin (0.2% BSA, supplied by the 10% FBS added to the culture media), or with albumin at sub-physiological (1.7%) or quasi-physiological (3.2%) concentrations. MAC-T cells were used for this experiment. Results are presented as fold change over vehicle control (0.5% DMSO). Asterisks indicate significant differences when compared to baseline (3.9 μmol/L NEFA), with each color corresponding to each BSA group. For each NEFA level, ψ denotes significant differences between 0.2 and 3.2% BSA; ϕ denotes significant differences between 0.2% and 1.7% BSA, and ω denotes significant differences between 1.7% and 3.2% BSA. The model-wise significance level of each parameter is presented in the figure. NEFA concentration on X-axis is presented on a logarithmic scale

To assess the effect of NEFA on PPAR activation, isolated NEFA from a pooled serum sample of five cows at 10 days postpartum were used to treat MAC-T cells in a dose-response assay (Fig. 1). Isolated NEFA activated PPAR in a dose-dependent manner starting at 100 μmol/L, with a linear response of the reporter up to the maximum we tested, i.e., 400 μmol/L. Due to their low solubility in aqueous solutions, circulating FA are transported bound to albumin, which may alter their uptake by peripheral tissues [33]. However, the addition of albumin at either sub-physiological (1.7%) or quasi-physiological (3.2%) concentrations significantly reduced the effect observed in DMEM (containing approx. 0.2% BSA from the 10% FBS added).

**Fig. 2:**
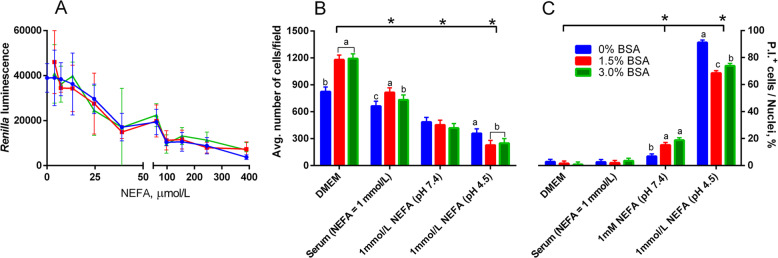
Cytotoxic effect of NEFA in MAC-T cells is pH-dependent and partly reduced by albumin added to the media. **a** Activity of constitutively expressed *renilla* luciferase decreases with increasing doses of NEFA isolated from a pool of blood serum from post-partum cows. Vertical bars represent standard error. **b** Cell proliferation, expressed as the average count of Hoescht 33342-positive cells per field imaged at 10×. Letters indicate model-wise differences; asterisks indicate significant differences when compared to DMEM control (*P* < 0.01). **c** Apoptosis measured via fluorescent imaging and expressed as a measure of propidium iodide positive cells over Hoescht 33342-positive cells. Letters indicate within-group differences; asterisks indicate significant differences when compared to DMEM control (*P* < 0.01)

Transition dairy cows are routinely exposed to circulating NEFA over 1 mmol/L in the few days after parturition [1]; thus, we were interested in exploring the effect of serum NEFA on PPAR activation past the 400 μmol/L threshold. When diluting isolated serum NEFA in culture media at equimolar concentrations to the one found in serum of early-postpartum cows, the high abundance of FA had a strong acidifying effect in the medium, decreasing the pH to 4.5 and killing the cells. This was observed through the activity of the constitutively expressed TK-driven *renilla* luciferase (Fig. 2a), which decreased as the concentration of NEFA in the culture medium increased. Constitutively expressed gene reporters, such as TK-driven *renilla* is a reliable indicator of cytotoxicity and apoptosis [34]. We further quantified via fluorescent imagining the cytotoxic effect after 24 h of culture, both from the perspective of reduction in cell proliferation (Fig. 2b) and of acute cytotoxicity (Fig. 2c). One mmol/L of isolated NEFA in buffered DMEM resulted in a strong decrease in pH reaching 4.5, and an almost total cellular death. While a positive effect of albumin in preventing cell death with 1 mmol/L isolated NEFA was observed, the loss in viability was still very high (approx. 80%). Adjusting the pH of the solution to a physiological level (i.e., 7.4) with sodium hydroxide reduced acute cytotoxicity; however, the percentage of apoptotic cells was still higher compared to MAC-T cells treated with only DMEM, suggesting that the cytotoxic effect of NEFA goes beyond the pH change. Further, cellular proliferation did not significantly increase in the buffered NEFA solution, with both buffered and unbuffered NEFA solutions having a greatly reduced number of cells per well compared to the control or serum, at any concentration of BSA. Additionally, increasing the pH of the NEFA solution with NaOH would generate an additional source of variation: the NaOH added to the NEFA samples, to bring their pH to physiological levels, could not be added to the control, as the limited buffering capacity of the culture media would be quickly exhausted, and the pH would increase above acceptable standards for cell culture (> 9) complicating the interpretation of the results.

Treating the cells with the serum sample from which the NEFA isolate was obtained (and thus, with 1 mmol/L of NEFA) did not significantly increase the percentage of apoptotic cells compared to DMEM without NEFA, nor did it significantly affect cellular proliferation at 24 h; additionally, treating cells with serum allowed for a more robust estimation of the true effect of NEFA in the animal as serum albumin, as well as all other serum proteins, are present at a physiological concentration. For the above reasons, we decided to perform all subsequent gene reporter assay experiments in serum, and to utilize the sample’s existent NEFA concentration as an explanatory variable, instead of using isolated NEFA.

### NEFA present in blood serum activate PPAR in a dose-dependent fashion

**Fig. 3:**
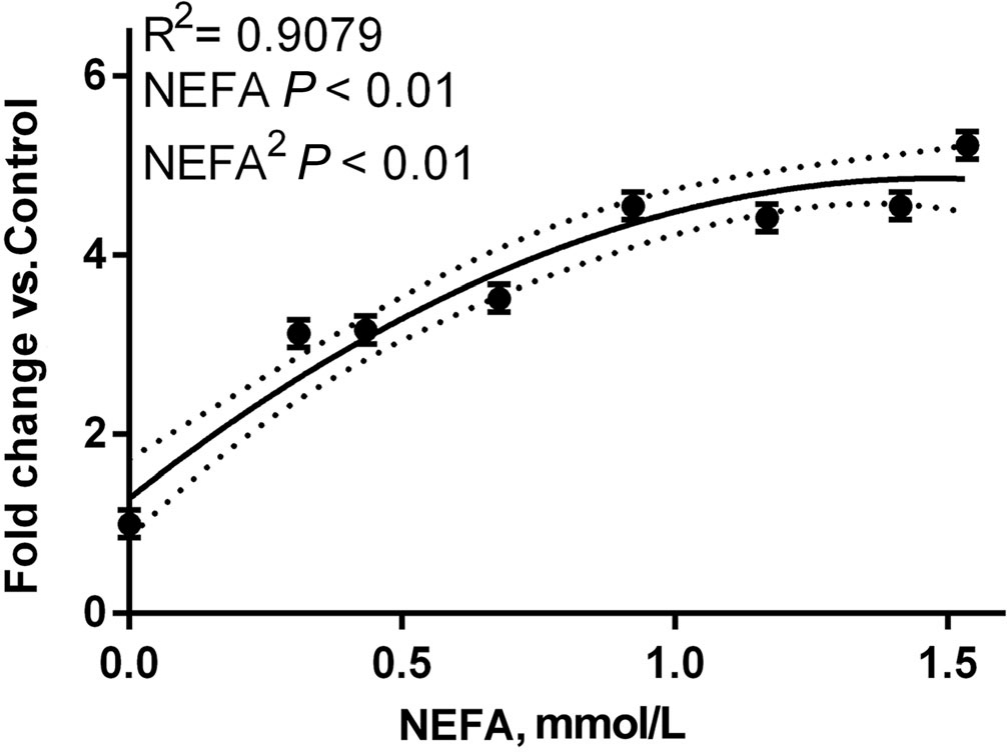
Activation of PPAR by NEFA in MAC-T cells is best represented by a quadratic model and is dose-dependent. PPAR activation measured using a 3xPPRE-TK-luciferase plasmid and normalized by co-transfected TK-renilla. Data are expressed as fold change over DMEM control (NEFA = 0 mmoL/l) by serum mixtures with different NEFA concentrations. The significance level of each parameter, as well as the R^2^ of the quadratic model are presented in the figure. Vertical bars indicate standard error

The treatment of MAC-T cells transfected with PPAR gene reporter with bovine blood serum containing increasing amount of NEFA had a quadratic effect on PPAR activation (Fig. 3). The response can be described by the following equation: *PPAR* = 4.834 × *NEFA* − 1.637 × *NEFA*^2^ + 1.286, where *PPAR* represents fold activation of the PPAR gene reporter relative to the DMEM control. The calculated maximum activation is obtained with NEFA concentration in blood serum of 1.474 mM. It is interesting to note that, in our model, PPAR activation reaches a plateau past its peak. This might partly be explained by the cytotoxic effect of NEFA discussed previously, which is supported by the negative correlation between *renilla* activity and NEFA concentration in the samples (Suppl. Figure 2). More importantly, the serum NEFA molarity required to achieve peak PPAR activation (~ 1.5 mM in our model) is characteristic of dairy cows in the early post-partum, which are known to present NEFA level between 1 and 2 mmol/L [1]. Most cows included in our experiment achieved a NEFA molarity > 0.7 mmol/L in the early postpartum, with one approaching close to 1.6 mmol/L (Suppl. Figure 3).

### Serum NEFA activate PPAR delta and gamma, but not PPAR alpha, in bovine mammary, endothelial and hepatic cells

**Fig. 4:**
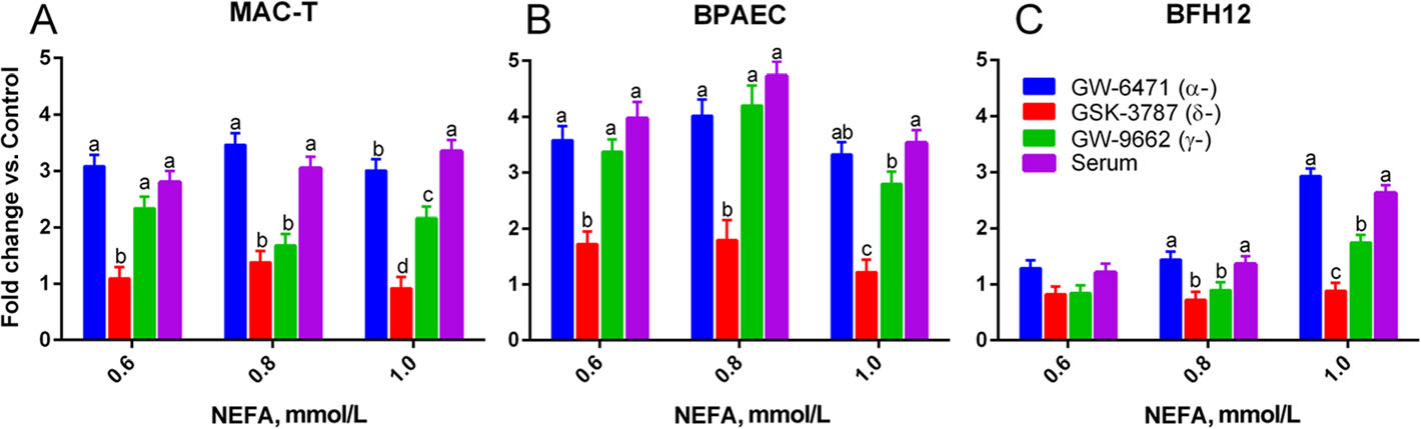
Serum NEFA activate PPARδ and, to a lesser extent, PPARγ. Effect of increasing serum NEFA molarity and PPAR isotype-specific antagonists on PPAR activation measured via gene reporter assay in mammary epithelial cells (MAC-T), **a**, bovine pulmonary artery endothelial cells (BPAEC), **b** and bovine liver cells (BFH-12), **c**. Letters indicate significant differences (*P* < 0.05) within each NEFA concentration. Results are presented as a ratio of each treatment over vehicle control (culture media + DMSO)

To elucidate the effect of serum NEFA on single PPAR isotypes, immortalized bovine cells modelling mammary (MAC-T), liver (BFH-12) and endothelial (BPAEC) cells, transfected with the PPAR gene reporter were treated with or without PPAR-specific antagonists for PPARα (GW-6171), PPARδ (GSK-3787) and PPARγ (GW-9662) with the dose that had the observed maximal inhibition of PPAR activity in MAC-T cells (Suppl. Figure 1). Serum NEFA activate PPARδ to the greatest extent, followed by PPARγ in MAC-T cells (Fig. 4a). The addition of the PPARα antagonist had no effect, with the only exception being the sample at 1 mmol/L NEFA, where a mild decrease in PPAR activation was observed. In bovine endothelial (BPAEC) cells no effect was detected on PPAR activity by the PPARα antagonist, an effect of the PPARγ antagonist only at 1 mmol/L NEFA, and an effect of the PPARδ antagonist at all concentrations of NEFA (Fig. 4b). Finally, BFH-12 cells, an immortalized model of bovine liver, had a decreased activity of the PPAR reporter following the application of the PPARδ and PPARγ antagonists, but no effect was measured after addition of the PPARα antagonist (Fig. 4c).

### Palmitate activates PPARδ and PPARα, but not PPARγ, in a dose-dependent manner

Similarly to the effect that negative energy balance has on the periparturient dairy cow, feed restriction causes a change in concentration and composition of the circulating NEFA [35]. In addition, it has been demonstrated that feeding saturated or unsaturated fat to pre-partum dairy cows alters the composition of serum NEFA, hepatic phospholipid fractions and liver triglycerides, without significantly increasing postpartum NEFA concentrations [36]. In this context, we were interested in assessing the effect of supplemental palmitate (C16:0), one of the major components of peripartum NEFA in dairy cows [36] and a common ingredient of lipid supplements used in dairy cows; additionally, palmitic acid has been shown to activate PPAR in bovine *in vitro* models [18, 37]. Thus, we deemed it relevant to investigate the effect of increasing doses of palmitate on the activation of PPAR in our MAC-T cells model.

**Fig. 5:**
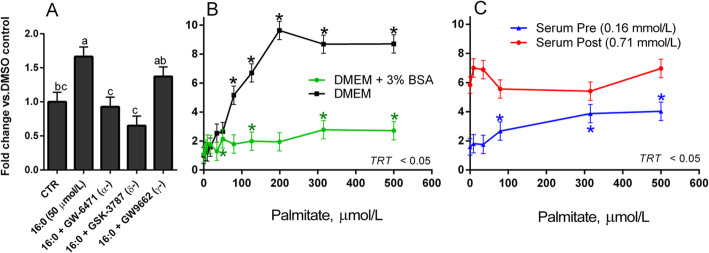
Palmitic acid activates PPARδ and PPARα, but not PPARγ, in a dose-dependent fashion. **a** Activation of individual PPAR isotypes by palmitic acid (16:0) in BFH-12 cells cultured in DMEM with 10% FBS. Different letters denote significant differences between treatments. GW-6471, PPARα antagonist; GSK-3787, PPARδ antagonist; GW-9662, PPARγ antagonist. **b** Effect on PPAR activation of an increasing dose of palmitic acid, when diluted in culture media with and without a quasi-physiological concentration of albumin (3%); treatment effect is indicated in the bottom-right corner of the panel. **c** Effect on PPAR activation of an increasing dose of palmitic acid added to serum samples from before and after parturition (Serum Pre, − 18 ± 15 DIM and Serum Post, 9 ± 2 DIM respectively). Asterisks indicate significant differences when compared to each treatment’s baseline (palmitic acid = 0 μmol/L). Treatment effect is indicated in the bottom-right corner of the panel

Palmitate activated PPAR up to ~ 10 fold when added to the culture media, unbound from albumin, in a dose-dependent manner (Fig. 5b). Similar to our results with isolated NEFA, the addition of quasi-physiological concentrations of albumin reduces activation of PPAR considerably (maximum activation ~ 2-fold). Interestingly, the activation of PPAR isotypes by palmitic acid did not fully resemble that of serum NEFA, as it significantly activated PPARδ and PPARα, but not PPARγ (Fig. 5a).

An additional point of interest was to investigate the effect on PPAR activation of supplementing palmitate in serum of cows collected during the transition period. For this purpose, we added increasing doses of palmitate to MAC-T cells cultured in pooled serum samples either before parturition, and therefore with low circulating NEFA (− 18 ± 15 DIM, 0.16 mmol/L NEFA, Fig. 5c, blue line), or in pooled serum samples from post-partum with high circulating NEFA (9 ± 2 DIM, 0.71 mmol/L NEFA, Fig. 5c, red line). While the activation of PPAR is greatest in the serum post-partum at baseline, likely due to the greater concentration of NEFA in the starting sample, the activity of the PPAR reporter is essentially unchanged at every concentration of palmitate up to 500 μmol/L. On the other hand, adding palmitate to the pre-partum serum sample resulted in a > 3-fold increase in PPAR activation, with a marked dose-dependent response.

These results offer grounds for two enticing ideas: first, supplementing palmitic acid may lead to activation of PPAR in the prepartum, but not in the postpartum, with a dose as low as ~ 100 μmol/L; second, palmitate supplementation activates PPARδ and PPARα, suggesting that PPAR activation by circulating NEFA might be guided by several different FA, and that targeted supplementation of specific FA could activate one or two PPAR isotypes preferentially. In early lactation, the liver and mammary gland take care of the bulk of FA metabolism in dairy cows [1]. Since most of our experiments concerned mammary (MAC-T) and liver (BFH-12) cells, it would be challenging to extend our finding to other organs. Our findings are also difficult to extend to other physiological stages and other FA, also considering differences in uptake of each FA. As an example, there is a large difference in mammary uptake of individual FA, ranging from ~ 16% of trans-C18:1 to almost 50% of C16:0 [38].

### Dietary fatty acids, released from VLDL activate PPAR in a dose-dependent manner

Supplementation of FA in dairy cows leads to increased concentrations of circulating FA [39]; however, this is likely due to release of NEFA from VLDL via lipoprotein lipase (LPL), since dietary long-chain FA are incorporated in VLDL by intestinal villi [40]. Previous authors have underlined that hydrolysis of lipoproteins by LPL induces a larger activation of PPAR compared to circulating NEFA, which has been attributed to the localized generation of large concentration of unbound FA [22]. This is of high importance for the possibility of inducing the expression of PPAR target genes via dietary approaches (i.e., nutrigenomics).

**Fig. 6:**
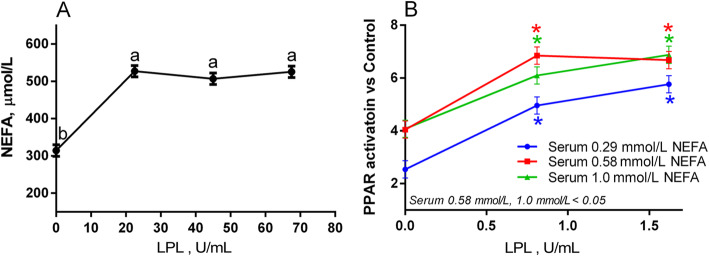
**a** NEFA concentration in serum from post-partum Jersey cows (11 ± 3 DIM) containing 0.32 mmol/L NEFA treated with physiological (22.5 U/mL) and supraphysiological (45 and 67.5 U/mL) concentrations of lipoprotein lipase for 24 h. **b** PPAR activation measured by gene reporter assay in MAC-T cells by serum samples with low (0.29 mmol/L), medium (0.58 mmol/L) and high (1.0 mmol/L) circulating NEFA, treated with two subphysiological, non-cytotoxic doses of LPL (0.81 and 1.62 U/mL). Asterisks reflect significant differences when compared with each group’s baseline (LPL = 0); colors refer to treatment group. Overall effect of treatments is indicated in the bottom-left corner of the panel

To elucidate whether the addition of LPL into a serum sample (i.e., containing VLDL) would generate an increase in the concentration of free FA in the samples, we treated a mixture of serum samples with 3 amounts of LPL from bovine milk: 22.5, 45.0 and 67.5 U/mL for 24 h at 37 °C (Fig. 6a). The low dose, resembling the activity measured *in vivo* in bovine plasma, was extrapolated from Ani et al. (2010) [41]. The addition of any amount of LPL generated a large release of free FA, increasing the FA concentration from 0.29 mmo/L up to over 0.5 mmo/L in all three treatments. However, due to the commercial formulation of the LPL used being diluted at relatively low concentrations (< 1 U/μL) in ammonium sulphate/TRIS-HCl, the amount required to achieve quasi-physiological concentrations was inherently toxic to the cells (Suppl. Figure 4A). Thus, we decided to decrease the concentrations of LPL by about 10-fold, adding only 1 μL of LPL/mL of serum and 2 μL of LPL/mL serum (0.81 and 1.62 U/mL, respectively), to three serum pools, at low (0.29 mmo/L), medium (0.58 mmo/L) and high (1.0 mmo/L) circulating NEFA. These concentrations proved to be suitable to treat the cells, with only a small (but significant) decrease in viability in the serum with low NEFA, measured by the intensity of *renilla* luminescence, and no significant effect on the other two groups (Suppl. Figure 4B). Both of these low concentrations of LPL (0.81 and 1.62 U/mL) effect a significant increase in PPAR activation in all three serum samples, ranging from 1.5- to 2-fold when compared to the untreated baseline, though with no significant difference in activation between the two concentrations of LPL. Only MAC-T cells were employed for this experiment, as there is sufficient evidence to underline a major role of LPL in the mammary gland of dairy cows [42], with a marked increase in the early postpartum, until peak lactation [43]. On the other hand, activity of LPL or hepatic lipase in the bovine liver is negligible [44], so exposing BFH-12 to serum+LPL mixtures would generate an environment of little relevance to the actual biology of the animal.

The above results appear to be concordant with the results from NEFA release by LPL in Fig. 6a, where all LPL treatments freed FA to the same extent regardless of concentration, and may be explained by the exhaustion of LPL substrate (in this case, VLDL) or by an inhibition of LPL by released NEFA [45]. The first explanation is supported by the known amount of TAG in blood of peripartum cows (approx. 100 nmol/mL, [46]) and the activity of the used LPL (1 unit releases one nmol of FA per minute). Assuming a constant activity of the LPL and 3 mol of FA for each mole of TAG, we would expect a complete release of all FA by the lowest dose used (0.81 U/mL) after just approx. 6 h; thus, all three concentrations would break down all the available TAG present in lipoproteins in the assayed time (24 h), at least from a theoretical standpoint. On the other hand, when measuring NEFA released with LPL at 0.81 or 1.6 U/mL (low doses), our results were conflicting: while a slight increase in NEFA concentration was detected with the serum with low NEFA (0.29 mmol/L), no change was measurable in the serum with medium NEFA (0.58 mmol/L), and the NEFA concentration in the serum sample with high NEFA (1 mmol/L) actually decreased with the addition of LPL (Suppl. Figure 5). The explanation for these results may be twofold: on the one hand, the limited sensitivity of the assay (14 μmol/L) might be too low to detect the release of NEFA at such low concentrations of LPL; on the other hand, as other researchers have detected [45, 47], increased circulating NEFA may inhibit the activity of LPL, as seems to be the case in our results. Nevertheless, the results of our assay suggest that even relatively small amounts of LPL can increase the local concentration of FA that the cells are exposed to, therefore significantly increasing PPAR activation.

## Discussion

Our results outline a tight-knit relationship between the metabolism of lipids and the activation of PPAR during the transition from pregnancy to parturition, highlighting a direct causal effect of free FA on PPAR activity. The role of PPAR in the biology of transition cows is still subject of intense study; a previously published hypothetical model of PPAR isotypes activation to aid the transition cows involved the activation of each PPAR isotype in different time to provide a possible benefit by activating PPAR targets [18]. Said model suggested a role of the PPAR network in several key genes involved in productive stages of lactating dairy cows, such as the involvement of PPARα and PPARδ in FA oxidation, the regulation of milk fat synthesis by PPARγ independently or through SREBP1, and the involvement of PPARα and PPARγ in regulating the inflammatory response.

Our results show that an increase of NEFA in blood serum activates PPARδ and PPARγ preferentially in both mammary and liver cells, with a marked quadratic dose-response. Others have reported increase of PPAR target genes such as pyruvate carboxylase [16, 48], *SLC27A1* [49], and others [50], when cows are fed-restricted, which might be caused by the activation of PPAR by increased concentration of NEFA. Expression of several of those genes and other putative PPAR target genes are known to be increased when PPAR agonists are added to culture with ruminant cells [18].

Other authors have noted that a restriction in feed intake prior to parturition improves the ability of the liver to metabolize TAG, reduces incidence of hepatic lipidosis, while not significantly increasing postpartum NEFA [51]. This could also be tied to an increase in PPAR activity, which in turn would increase FA oxidation rates earlier in the peripartum by activating PPARα and PPARδ, would decrease lipolysis by increasing insulin sensitivity in adipose tissue by activating PPARγ, and improve liver ability to deal with metabolic challenges in the postpartum. Our results of a similar activation of PPAR isotypes in bovine mammary and liver cells appear to indicate a possible simultaneous increase in expression of similar genes in both organs. However, the activation of PPAR in mammary cells was more pronounced at lower doses compared to liver cells. This, together with the fact that the liver uptakes NEFA at a constant rate [1] while the mammary tissue has increased uptake of NEFA early post-partum [52], might indicate a larger effect of PPAR by NEFA on mammary tissue vs. liver.

The predominant activation of PPARδ by serum isolated from cows during the transition period is somewhat surprising. Studies of the transcriptome of transition dairy cows [16] suggested a major role of PPARα in the transition from pregnancy to lactation, especially in the liver, where this isoform has the highest expression of the three [18]. Nevertheless, transcriptomic studies of cows undergoing negative energy balance, either during the transition period or as a result of dietary management [50], reveal the modulation of several genes shown to be regulated by PPARδ in other species, such as *Lpin1* and *Slc71a1*, which responded to the PPARδ agonist GW-50156 in mice [53], or *Ppargc1a*, which was upregulated through PPARδ by treatment with GW-50156 in C2C12 mouse myogenic cells [54]. Interestingly, the latter study found that the expression of *Ppargc1a*, and the regulation of oxidative metabolism controlled by it, were not mediated by PPARα, suggesting that control of FA oxidation independent from PPARα is possible. Finally, others have underlined the role of PPARδ, and not of PPARα, in sensing and responding to circulating free FA in non-ruminants [23, 55].

Another key finding outlined in our experiments is the preferential activation of PPARα and PPARδ, but not PPARγ, by palmitic acid added in culture. This is in stark contrast with our findings with serum NEFA, where PPARδ and PPARγ were found to be activated, but not PPARα. Our results suggest that individual FA preferentially activate one or two PPAR isotype, and that specific dietary FA (or a mixture of FA) may be used to target a specific isotype. This, combined with our finding that additional palmitate to postpartum serum containing high NEFA serum did not increase further PPAR activation, makes a case for fat supplementation in prepartum dairy cows, when NEFA levels are low. Previous experiment indicated a reduction of feed intake post-partum [56] or no effect on milk yield and liver lipid accumulation [57, 58] or an even worse metabolic situation post-partum [59] by supplementing saturated fat pre-partum in dairy cows. However, other studies support a priming of the cows’ metabolism to better perform during the early post-partum, including better reproductive performance, when supplemented prepartum with saturated (mainly C16:0 and C18:0) [24] or unsaturated FA [60–62]. Thus, our data appear somewhat in contrast to* in vivo* data. However, except for one of the above cited studies, none focused on PPAR activation, and none provided data suggesting that the dose and/or mixture of FA used could effectively activate PPAR. Furthermore, supplementation of palmitic acid generally improves the health and performance of dairy cows [63, 64] but high dose of palmitic acid can also decrease cell viability and increase inflammation by both binding to TLR2 or TLR4 and increasing ceramide production [65, 66]. Thus, it is important to carry out more studies to determine the dose that can optimize PPAR activation but minimize inflammation, especially* in vivo*.

Our results recapitulate the role of albumin in mediating the response of PPAR to FA as previously reported [18], both when added to culture media (as presented in Figs. 1 and 5b) and when adding FA to blood serum, which contains physiological concentrations of albumin (Fig. 5c). This is especially relevant considering that the solubility of most FA in aqueous solutions is below 50 μmol/L at physiological pH with palmitic acid having a solubility of 10^− 10^ mol/L [67], and that NEFA mostly circulate bound to albumin [33]; this would seem to indicate that *in vivo* activation of PPAR by FA would require much higher molarities than those presented in Figs. 1 and 5b. The impact of albumin on PPAR activation in reporter assays has been previously studied [22, 68], and is generally attributed to its ability to reversibly bind FA [69] and render them inaccessible to PPAR. While the concentration of serum albumin is known to fluctuate during the transition period, the degree of said change is generally within 10 g/L (i.e, 3% to 4% or 30 to 40 g/L) [13]. However, speculating upon the quantitative relevance of our finding is challenging, as the use of DMEM with 10% FBS which was the conditions of our *in vitro* experiment do not resemble those encountered in the organism. Indeed, it has been postulated that the most likely mechanism of absorption of albumin-FA complexes is through clefts in the fenestrated endothelium (such as in the liver), where binding of albumin to the receptor albondin causes dissociation of the albumin-FA complex, and subsequent uptake of the FA [33]. It is unclear if this process did happen at all in our cell model. Additionally, the albumin utilized in our assays was completely devoid of FA, which is likely not the case for most circulating albumin in a physiological state. Thus, it is likely that the sequestering capacity of albumin for FA in our cell culture was higher than what expected *in vivo*. Based on above considerations, more research is needed to determine the impact of circulating albumin in the availability of NEFA as PPAR ligand. The use of the hybrid model in some of our experiments was done to overcome the limitations associated with the use of albumin and better mimic the* in vivo* situation, but such model does not allow to study with precision the role of albumin in regulating FA availability for PPAR.

Taken together, our results suggest a two-way approach to practical nutrigenomics in dairy cows:

1. early supplementation of FA, targeting PPAR isotypes of interest, can prime the animal to respond better to the metabolic challenges of the early post-partum. The priming of the liver by FA is mainly supported by the transcriptomic data of liver from cows fed-restricted in the last phase of pregnancy that had higher circulating NEFA [17]. Being NEFA composed of several FA, results of that study suggest that a mixture of FA might be more effective in activating PPAR than a single FA. However, such inference requires further studies with the aim to identify the type and dose of FA or a mixture of FA that can target each PPAR isotype.
2. On the other hand, supplementing dairy cows pre-partum with specific FA can alter the FA composition of the adipose tissue, allowing to enrich PPAR-targeting FA to be released later in the form on NEFA [36].

While this ambitious model is far from validated, and further information is needed on the effect of dietary fat and individual FA on PPAR activation *in vivo*, it offers us some insight of what may be considered a novel approach to ruminant nutrition, one that focuses on fine-tuning metabolism through nutrigenomic mechanisms.

### Limitations of the study

The experiments were carried out under the assumption that PPAR antagonists are specific to the isotype they are meant to target, and do not affect the activity of the other two. While this assumption is known to be true for non-ruminants, peculiarities of the bovine PPAR might alter this. Further, while a large portion of the activation of PPAR was explained by the concentration of NEFA in the serum samples, it is also entirely possible that other factors be at play, considering the large number of components present in serum.

In the present study we did not measure uptake of FA or intracellular trafficking of FA. Proper assessment of FA uptake and trafficking would have helped to better understand which FA is taken up by cells and which FA translocate into the nucleus for PPAR activation. Although overall uptake of FA is relatively easy to assess (i.e., measurement of NEFA in culture media through time as performed previously [37]), the uptake and intracellular trafficking of specific FA would involve the ability to track specific FA (e.g. with fluorescence or radiolabeling). Capacity to import FA can be also inferred by the expression of genes and/or proteins related to FA import; however, this was not assessed in the present study.

Finally, some of the variation in single PPAR activation may have been explained by the expression profile of PPAR in our models, which cannot be controlled for. Despite all the above limitations, the assays reported in this manuscript was enough to achieve the goal as our intention was a proof-of-concept of the direct activation of PPAR by serum NEFA.

## Conclusions

In this study, we demonstrated a direct causal link between circulating NEFA concentration and PPAR activation, using a hybrid *in vivo-in vitro* model, where whole blood serum was combined with cells to ensure a more representative biological environment. Further, we showed increased PPAR activation upon palmitate addition to DMEM and serum of pre-partum cows and by the localized released of FA from lipoproteins (mainly derived from the diet) by LPL. Our results show that the presence of albumin at physiological concentrations limits the agonistic capacity of FA and reduces activation of PPAR.

When translating our results to *in vivo* nutrigenomics approach, an important finding was the response of PPAR to NEFA only pre-partum, indicating the need to scheduled FA supplementation at a time when circulating NEFA are not at their peak concentration in plasma, i.e. before (not after) parturition. A logical progression to our findings is to validate our observation *in vivo*, by studying the effect of individual and combined FA, supplemented through the diet, on PPAR activation in dairy cows.

## Supplementary information

**Additional file 1 : Suppl. Figure 1.** Identification of optimal concentration for individual PPAR antagonists. Compounds are added to serum at increasing concentrations. Results are presented as a ratio of the uninhibited baseline (serum only). Different letters indicate significant differences within a group (*P* < 0.05). Optimal concentration was determined using a 4-point linear dilution where the lowest concentration was set at 500 nmol/L, and the highest was set at double the typical concentration found in the literature. For both GW-6471 and GW-9662, the four concentrations used were 500 nmol/L, 33.67 μmol/L, 66.83 μmol/L and 100 μmol/L. For GSK-3787, the concentrations were 500 nmol/L, 3.37 μmol/L, 6.68 μmol/L and 10 μmol/L.

**Additional file 2 : Suppl. Figure 2.** NEFA in serum mixtures is mildly cytotoxic. Correlation between serum NEFA concentration and *renilla* luminescence. Correlation coefficient (r) and significance level are indicated in the figure.

**Additional file 3 : Suppl. Figure 3.** Serum NEFA concentrations of the five animals included in the study during the experimental period. DIM = days in milk, days relative to parturition.

**Additional file 4 : Suppl. Figure 4. A)** Cytotoxicity of NEFA released with the addition of physiological and supraphysiological concentrations of LPL, measured relative to the change in *renilla* luminescence. Results are presented as raw luminescence units. Error bars represent standard error. **B)** Cytotoxicity of NEFA released with the addition of sub-physiological concentrations of LPL to three serum samples with different starting NEFA concentrations, measured relative to the change in *renilla* luminescence. Results are presented as raw luminescence units. Asterisks refer to significant differences compared to baseline (LPL = 0), with the color referring to the specific group.

**Additional file 5 : Suppl. Figure 5.** Release of NEFA in three different serum pools, when treated with two sub-physiological, non-cytotoxic doses of LPL (0.81 and 1.62 U/mL). Letters refer to within group (serum) significant differences.

## Data Availability

The datasets used and/or analysed during the current study are available from the corresponding author on reasonable request.

## Abbreviations

BPAEC: Bovine pulmonary artery endothelial cells
BFH-12: Immortalized bovine fetal hepatocytes
DIM: Day in milk
FA: Fatty acid(s)
DMEM: Dulbecco’s Modified Eagle Media
FABP: Fatty acid-binding protein
LCFA: Long-chain fatty acid(s)
LPL: Lipoprotein lipase
MAC-T: Immortalized mammary alveolar cells
NEB: Negative energy balance
NEFA: Non-esterified fatty acids
PPAR: Peroxisome proliferator-activated receptor
PPRE: Peroxisome proliferator-activated receptor response element
TAG: Triacylglycerol
VLDL: Very-low density lipoproteins

## References

[1] Drackley JK (1999). Biology of dairy cows during the transition period: the final frontier?. J Dairy Sci.

[2] Drackley JK, Overton TR, Douglas GN. Adaptations of glucose and long-chain fatty Acid metabolism in liver of dairy cows during the periparturient period. J Dairy Sci. 2001;84:E100–12.

[3] Grummer RR (1995). Impact of changes in organic nutrient metabolism on feeding the transition dairy cow. J Anim Sci.

[4] Bradford BJ, Yuan K, Farney JK, Mamedova LK, Carpenter AJ (2015). Invited review: inflammation during the transition to lactation: new adventures with an old flame. J Dairy Sci.

[5] Seifi HA, Gorji-Dooz M, Mohri M, Dalir-Naghadeh B, Farzaneh N (2007). Variations of energy-related biochemical metabolites during transition period in dairy cows. Comp Clin Pathol.

[6] McArt JAA, Nydam DV, Oetzel GR, Overton TR, Ospina PA (2013). Elevated non-esterified fatty acids and β-hydroxybutyrate and their association with transition dairy cow performance. Vet J.

[7] Adewuyi AA, Gruys E, van Eerdenburg FJCM (2005). Non esterified fatty acids (NEFA) in dairy cattle. A review. Vet Q.

[8] Vazquez-Añon M, Bertics S, Luck M, Grummer RR, Pinheiro J (1994). Peripartum liver triglyceride and plasma metabolites in dairy cows. J Dairy Sci.

[9] Bobe G, Young JW, Beitz DC. Invited review: pathology, etiology, prevention, and treatment of fatty liver in dairy cows. J Dairy Sci. 2004;87:3105–24. 10.3168/jds.S0022-0302(04)73446-3 15377589

[10] Contreras GA, Sordillo LM (2011). Lipid mobilization and inflammatory responses during the transition period of dairy cows. Comp Immunol Microbiol Infect Dis.

[11] Drackley JK, Dann HM, Douglas N, Guretzky NAJ, Litherland NB, Underwood JP (2005). Physiological and pathological adaptations in dairy cows that may increase susceptibility to periparturient diseases and disorders. Ital J Anim Sci.

[12] Wankhade PR, Manimaran A, Kumaresan A, Jeyakumar S, Ramesha KP, Sejian V (2017). Metabolic and immunological changes in transition dairy cows: a review. Vet World.

[13] Bionaz M, Trevisi E, Calamari L, Librandi F, Ferrari A, Bertoni G (2007). Plasma Paraoxonase, health, inflammatory conditions, and liver function in transition dairy cows. J Dairy Sci.

[14] Contreras GA, Strieder-Barboza C, Koster JD (2018). Symposium review: modulating adipose tissue lipolysis and remodeling to improve immune function during the transition period and early lactation of dairy cows. J Dairy Sci.

[15] Janovick NA, Boisclair YR, Drackley JK (2011). Prepartum dietary energy intake affects metabolism and health during the periparturient period in primiparous and multiparous Holstein cows1. J Dairy Sci.

[16] Loor JJ, Dann HM, Guretzky NAJ, Everts RE, Oliveira R, Green CA (2006). Plane of nutrition prepartum alters hepatic gene expression and function in dairy cows as assessed by longitudinal transcript and metabolic profiling. Physiol Genomics.

[17] Shahzad K, Bionaz M, Trevisi E, Bertoni G, Rodriguez-Zas SL, Loor JJ (2014). Integrative analyses of hepatic differentially expressed genes and blood biomarkers during the Peripartal period between dairy cows overfed or restricted-fed energy Prepartum. PLoS One.

[18] Bionaz M, Chen S, Khan MJ, Loor JJ. Functional role of PPARs in ruminants: potential targets for fine-tuning metabolism during growth and lactation. PPAR Research. 2013. 10.1155/2013/684159. 10.1155/2013/684159 PMC3657398 23737762

[19] Janani C, Ranjitha Kumari BD (2015). PPAR gamma gene – A review. Diabetes Metab Syndr.

[20] Nakamura MT, Yudell BE, Loor JJ (2014). Regulation of energy metabolism by long-chain fatty acids. Prog Lipid Res.

[21] Kersten S, Seydoux J, Peters JM, Gonzalez FJ, Desvergne B, Wahli W (1999). Peroxisome proliferator–activated receptor α mediates the adaptive response to fasting. J Clin Invest.

[22] Ruby MA, Goldenson B, Orasanu G, Johnston TP, Plutzky J, Krauss RM (2010). VLDL hydrolysis by LPL activates PPAR-α through generation of unbound fatty acids. J Lipid Res.

[23] Sanderson LM, Degenhardt T, Koppen A, Kalkhoven E, Desvergne B, Müller M, et al. Peroxisome proliferator-activated receptor β/δ (PPARβ/δ) but not PPARα serves as a plasma free fatty acid sensor in liver. Mol Cell Biol. 2009;29:6257–67. 10.1128/MCB.00370-09 PMC2786701 19805517

[24] Akbar H, Schmitt E, Ballou MA, Corrêa MN, DePeters EJ, Loor JJ. Dietary lipid during late-pregnancy and early-lactation to manipulate metabolic and inflammatory gene network expression in dairy cattle liver with a focus on PPARs. Gene Regul Syst Bio. 2013;7:GRSB.S12005. 10.4137/GRSB.S12005 PMC3699062 23825924

[25] Mottillo EP, Bloch AE, Leff T, Granneman JG (2012). Lipolytic products activate peroxisome proliferator-activated receptor (PPAR) α and δ in brown adipocytes to match fatty acid oxidation with supply. J Biol Chem.

[26] Jaaf S, Batty B, Krueger A, Estill C, Bionaz M. Selenium biofortified alfalfa hay fed in low quantities improves selenium status and glutathione peroxidase activity in transition dairy cows and their calves. J Dairy Res. 2020;87(2):184–90. 10.1017/S002202992000028X. 10.1017/S002202992000028X 32295653

[27] Muraglia A, Nguyen VT, Nardini M, Mogni M, Coviello D, Dozin B, et al. Culture medium supplements derived from human platelet and plasma: cell commitment and proliferation support. Front Bioeng Biotechnol. 2017;5:66. 10.3389/fbioe.2017.00066. 10.3389/fbioe.2017.00066 PMC5702080 29209609

[28] Contreras GA, O’Boyle NJ, Herdt TH, Sordillo LM (2010). Lipomobilization in periparturient dairy cows influences the composition of plasma nonesterified fatty acids and leukocyte phospholipid fatty acids. J Dairy Sci..

[29] Gleich A, Kaiser B, Schumann J, Fuhrmann H (2016). Establishment and characterisation of a novel bovine SV40 large T-antigen-transduced foetal hepatocyte-derived cell line. In Vitro Cell Dev Biol-Anim..

[30] Carpenter AE, Jones TR, Lamprecht MR, Clarke C, Kang IH, Friman O (2006). CellProfiler: image analysis software for identifying and quantifying cell phenotypes. Genome Biol.

[31] Kim JB, Wright HM, Wright M, Spiegelman BM (1998). ADD1/SREBP1 activates PPARgamma through the production of endogenous ligand. Proc Natl Acad Sci U S A.

[32] Osorio JS, Bionaz M (2017). Plasmid transfection in bovine cells: optimization using a realtime monitoring of green fluorescent protein and effect on gene reporter assay. Gene..

[33] van der Vusse GJ (2009). Albumin as fatty acid transporter. Drug Metab Pharmacokinet.

[34] Paguio A, Stecha P, Wood KV, Fan F (2010). Improved dual-luciferase reporter assays for nuclear receptors. Curr Chem Genomics.

[35] Rukkwamsuk T, Geelen MJH, Kruip TAM, Wensing T. Interrelation of fatty acid composition in adipose tissue, serum, and liver of dairy cows during the development of fatty liver Postpartum1. J Dairy Sci. 2000;83:52–9. 10.3168/jds.S0022-0302(00)74854-5 10659963

[36] Douglas GN, Rehage J, Beaulieu AD, Bahaa AO, Drackley JK. Prepartum nutrition alters fatty acid composition in plasma, adipose tissue, and liver lipids of Periparturient dairy cows. J Dairy Sci. 2007;90:2941–59. 10.3168/jds.2006-225 17517735

[37] Thering BJ, Bionaz M, Loor JJ (2009). Long-chain fatty acid effects on peroxisome proliferator-activated receptor-α-regulated genes in Madin-Darby bovine kidney cells: optimization of culture conditions using palmitate. J Dairy Sci.

[38] Moate PJ, Chalupa W, Boston RC, Lean IJ (2008). Milk fatty acids II: prediction of the production of individual fatty acids in bovine Milk. J Dairy Sci.

[39] Andersen JB, Ridder C, Larsen T (2008). Priming the cow for mobilization in the Periparturient period: effects of supplementing the dry cow with saturated fat or linseed. J Dairy Sci.

[40] Bauman DE, Lock AL. Concepts in lipid digestion and metabolism in dairy cows. Proceedings of the 2006 Tri-State Dairy Nutrition Conference, Fort Wayne, Indiana, USA, 25–26 April, 2006. 2006;1–14.

[41] Ani A, Ani M, Moshtaghie A-A, Ahmadvand H (2010). Effect of titanium on lipoprotein lipase activity in vivo and in vitro. J Trace Elem Med Biol..

[42] Askew EW, Emery RS, Thomas JW (1970). Lipoprotein lipase of the bovine mammary gland. J Dairy Sci.

[43] Bionaz M, Loor JJ (2008). Gene networks driving bovine milk fat synthesis during the lactation cycle. BMC Genomics.

[44] Cordle SR, Yeaman SJ, Clegg RA (1983). Salt-resistant (hepatic) lipase: evidence for its presence in bovine liver and adrenal cortex. Biochim Biophys Acta.

[45] Peterson J, Bihain BE, Bengtsson-Olivecrona G, Deckelbaum RJ, Carpentier YA, Olivecrona T (1990). Fatty acid control of lipoprotein lipase: a link between energy metabolism and lipid transport. Proc Natl Acad Sci U S A.

[46] Graugnard DE, Bionaz M, Trevisi E, Moyes KM, Salak-Johnson JL, Wallace RL (2012). Blood immunometabolic indices and polymorphonuclear neutrophil function in peripartum dairy cows are altered by level of dietary energy prepartum1. J Dairy Sci.

[47] Saxena U, Witte LD, Goldberg IJ (1989). Release of endothelial cell lipoprotein lipase by plasma lipoproteins and free fatty acids. J Biol Chem.

[48] Velez JC, Donkin SS. Feed restriction induces pyruvate carboxylase but not phosphoenolpyruvate carboxykinase in dairy cows. J Dairy Sci. 2005;88:2938–48. 10.3168/jds.S0022-0302(05)72974-X 16027208

[49] Gross B, Pawlak M, Lefebvre P, Staels B (2017). PPARs in obesity-induced T2DM, dyslipidaemia and NAFLD. Nat Rev Endocrinol.

[50] Loor JJ, Everts RE, Bionaz M, Dann HM, Morin DE, Oliveira R (2007). Nutrition-induced ketosis alters metabolic and signaling gene networks in liver of periparturient dairy cows. Physiol Genomics.

[51] Dann HM, Litherland NB, Underwood JP, Bionaz M, D’Angelo A, McFadden JW (2006). Diets during far-off and close-up dry periods affect periparturient metabolism and lactation in multiparous cows. J Dairy Sci.

[52] Miller PS, Reis BL, Calvert CC, DePeters EJ, Baldwin RL. Patterns of nutrient uptake by the mammary glands of lactating dairy cows. J Dairy Sci. 1991;74:3791–9. 10.3168/jds.S0022-0302(91)78571-8 1757621

[53] Narkar VA, Downes M, Yu RT, Embler E, Wang Y-X, Banayo E (2008). AMPK and PPARdelta agonists are exercise mimetics. Cell..

[54] Hondares E, Pineda-Torra I, Iglesias R, Staels B, Villarroya F, Giralt M (2007). PPARδ, but not PPARα, activates PGC-1α gene transcription in muscle. Biochem Biophys Res Commun.

[55] Ravnskjaer K, Frigerio F, Boergesen M, Nielsen T, Maechler P, Mandrup S (2010). PPARδ is a fatty acid sensor that enhances mitochondrial oxidation in insulin-secreting cells and protects against fatty acid-induced dysfunction. J Lipid Res.

[56] Palmquist DL, Jenkins TC (2017). A 100-year review: fat feeding of dairy cows. J Dairy Sci.

[57] Duske K, Hammon HM, Langhof A-K, Bellmann O, Losand B, Nürnberg K (2009). Metabolism and lactation performance in dairy cows fed a diet containing rumen-protected fat during the last twelve weeks of gestation. J Dairy Sci.

[58] Salfer JA, Linn JG, Otterby DE, Hansen WP, Johnson DG. Early lactation responses of Holstein cows fed a rumen-inert fat prepartum, postpartum, or both. J Dairy Sci. 1995;78:368–77. 10.3168/jds.S0022-0302(95)76645-0 7745157

[59] Moallem U, Katz M, Arieli A, Lehrer H (2007). Effects of Peripartum propylene glycol or fats differing in fatty Acid profiles on feed intake, production, and plasma metabolites in dairy cows. J Dairy Sci.

[60] Colazo MG, Hayirli A, Doepel L, Ambrose DJ (2009). Reproductive performance of dairy cows is influenced by prepartum feed restriction and dietary fatty acid source. J Dairy Sci.

[61] Damgaard BM, Weisbjerg MR, Larsen T (2013). Priming the cow for lactation by rapeseed supplementation in the dry period. J Dairy Sci.

[62] Jolazadeh AR, Mohammadabadi T, Dehghan-banadaky M, Chaji M, Garcia M (2019). Effect of supplementing calcium salts of n-3 and n-6 fatty acid to pregnant nonlactating cows on colostrum composition, milk yield, and reproductive performance of dairy cows. Anim Feed Sci Technol.

[63] Loften JR, Linn JG, Drackley JK, Jenkins TC, Soderholm CG, Kertz AF (2014). Invited review: Palmitic and stearic acid metabolism in lactating dairy cows. J Dairy Sci.

[64] Guiling M, Merrill C, Kung L, Gressley TF, Harrison JH, Block E (2017). Effect of source of supplemental fat in early lactation on productive performance and milk composition. Prof Ani Sci.

[65] Rico JE, Mathews AT, Lovett J, Haughey NJ, McFadden JW (2016). Palmitic acid feeding increases ceramide supply in association with increased milk yield, circulating nonesterified fatty acids, and adipose tissue responsiveness to a glucose challenge. J Dairy Sci.

[66] Li P, Li L, Zhang C, Cheng X, Zhang Y, Guo Y, et al. Palmitic acid and β-hydroxybutyrate induce inflammatory responses in bovine endometrial cells by activating oxidative stress-mediated NF-κB signaling. Molecules. 2019;24:2421. 10.3390/molecules24132421 PMC6650895 31266188

[67] Vorum H, Brodersen R, Kragh-Hansen U, Pedersen AO (1992). Solubility of long-chain fatty acids in phosphate buffer at pH 7.4. Biochim Biophys Acta.

[68] Clarke HJ, Gregoire F, Ma F, Martin R, Zhao S, Lavan BE. Cross-species differential plasma protein binding of MBX-102/JNJ39659100: a novel PPAR-gamma agonist. PPAR Res. 2008;2008:465715. 10.1155/2008/465715. 10.1155/2008/465715 PMC2535826 18815616

[69] Spector AA (1975). Fatty acid binding to plasma albumin. J Lipid Res.

