# Supplementary figures and images for: The interplay between non-esterified fatty acids and bovine peroxisome proliferator-activated receptors: results of an in vitro hybrid approach
Source: J Anim Sci Biotechnol. 2020 Aug 11;11:91. doi: 10.1186/s40104-020-00481-y (PMC7419192; doi:10.1186/s40104-020-00481-y)

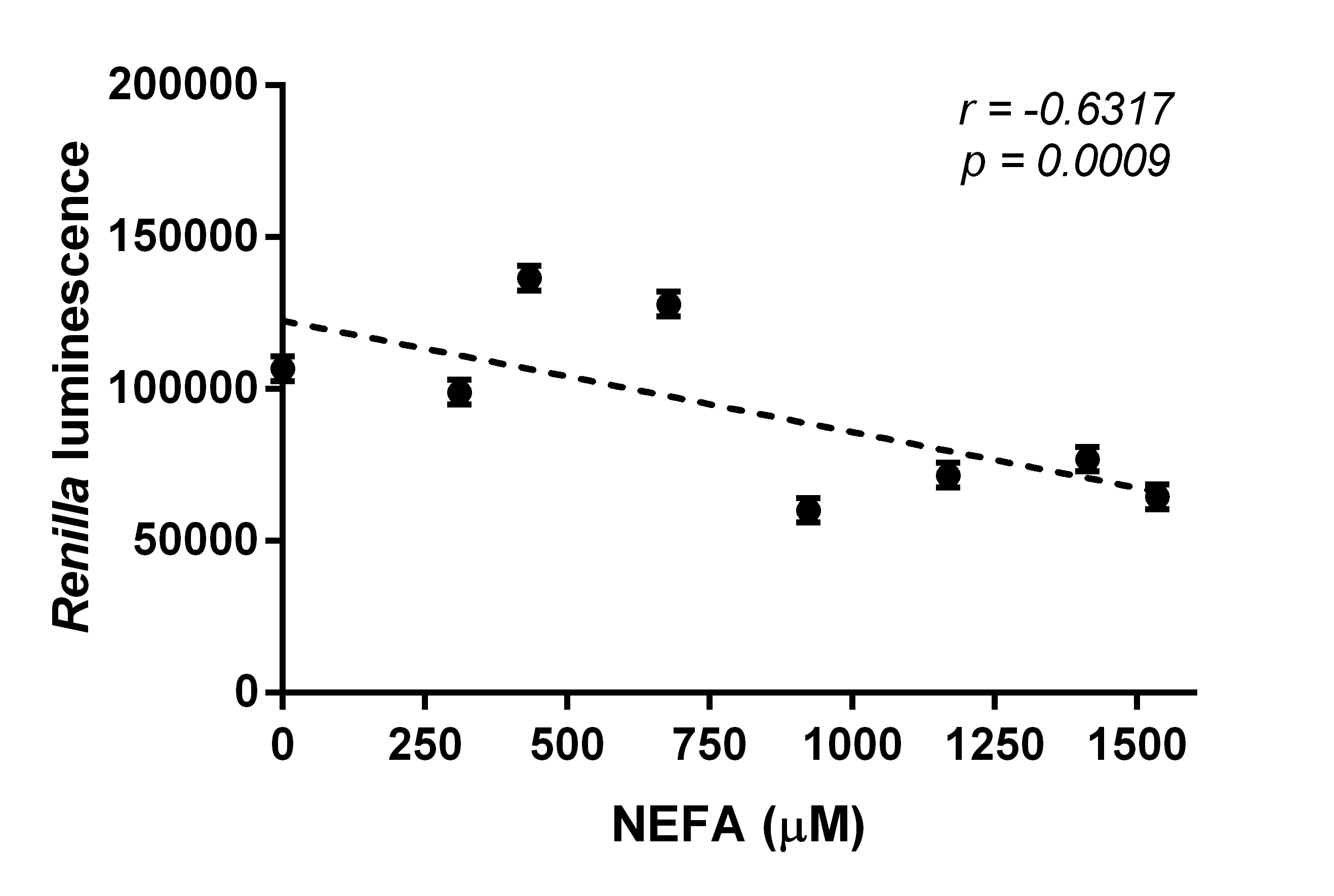

Supplement: Supplementary file 2 — Additional file 2 : Suppl. Figure 2. NEFA in serum mixtures is mildly cytotoxic. Correlation between serum NEFA concentration and renilla luminescence. Correlation coefficient (r) and significance level are indicated in the figure. [file 40104_2020_481_MOESM2_ESM.png]
